# Changes in Diversity Due to Long-Term Management in a High Natural Value Grassland

**DOI:** 10.3390/plants10040739

**Published:** 2021-04-09

**Authors:** Ioana Vaida, Florin Păcurar, Ioan Rotar, Liviu Tomoș, Vlad Stoian

**Affiliations:** 1 Department of Grasslands and Forage Crops, Faculty of Agriculture, University of Agricultural Sciences and Veterinary Medicine Cluj-Napoca, Calea Mănăştur 3-5, 400372 Cluj-Napoca, Romania; ioana.vaida@usamvcluj.ro (I.V.); ioan.rotar@usamvcluj.ro (I.R.); ltomos@yahoo.com (L.T.); 2 Department of Microbiology, Faculty of Agriculture, University of Agricultural Sciences and Veterinary Medicine Cluj-Napoca, Calea Mănăştur 3-5, 400372 Cluj-Napoca, Romania

**Keywords:** indicator species, agro-ecological spectrum, phytodiversity, long-term observations, fertilizer gradient

## Abstract

High nature value (HNV) grassland systems are increasingly important for the ecosystem services they provide and for their socio-economic impact in the current constant-changing context. The aim of our paper is to evaluate the long-term effect of organic fertilizers on HNV systems in the Apuseni Mountains, Romania. As an objective we want to identify the optimal intensity of conservation management and its recognition based on indicator value plant species. The experiments were established in 2001 on the boreal floor and analyze the effect of a gradient of four organic treatments with manure. Fertilization with 10 t ha^−1^ manure ensures an increase in yield and has a small influence on diversity, and could be a real possibility for the maintenance and sustainable use of HNV. Each fertilization treatment determined species with indicator value that are very useful in the identification and management of HNV. The indicator species are useful in forecasting the fluctuations and successions in grasslands, determined by the modification of the dominance-codominance ratio and the real coverage of each species.

## 1. Introduction

The ecosystem services of grasslands are the subject of an increasing number of studies nowadays, and have been classified and summarized in various ways. Grasslands provide four ecosystem services, which can be distinguished by their role: support, supply, regulation, and crop [1]. Semi-natural grasslands are increasingly appreciated for the ecosystem services they offer, such as unique biodiversity, carbon sequestration, water retention, heritage, and low fire risk [2]. Depending on the intensity of the applied management, grassland systems can influence climatic conditions both positively and negatively. Therefore, the promotion of smart farming practices is essential minimizing and regulating climate change and greenhouse gases [3]. Grassland ecosystems are able to tolerate quite well the more frequent periods of drought in recent years. There is currently a social interest in benefiting from the effects of biodiversity on the grassland ecosystems functioning to sustainable agriculture practice [4]. The ability of grasslands to provide multiple ecosystem services depends to a large extent of the applied management intensity [5]. Common agricultural policy promotes the maintenance of natural habitats and biodiversity, taking into account the economic, social, cultural and regional requirements as a contribution to the general objective of sustainable development [6]. The EU Habitats directive lists habitats that the member states must protect through the designation and management of protected areas known as Special Areas of Conservation [7]. At the European level, large grassland areas (30% of the total agricultural area) have been declared grasslands with high natural value [8]. HNV systems combine the maintenance of biodiversity plant community (included habitats 92/43 EEC) and productivity, but only up to a certain level of harvest [9]. Particular attention must be paid to the aspect of the pasture, and from a conservation point of view, the adoption of a grazing plan for the conservation of natural habitats [10], and of species of conservation interest, is crucial, while, in fact, with overgrazing, the populations of threatened plants may be numerically reduced until their extinction [11,12,13]. However, they remain important sources of feed and forage for the livestock. From a total number of almost 4,800,000 ha of grasslands in Romania, 2,000,000 ha are grasslands with high natural value, and these are mostly located along the Carpathian mountains [14]. In Romania, an important area of HNV systems has been maintained, compared to Austria, where in the last 60 years 50% of the area of semi-natural grasslands with high biodiversity has disappeared [15]. In general, the average levels of land use intensity in Eastern European areas are much lower than in Central and Northern Europe [16]. Agri-environmental schemes in post-communist countries have conserved semi-natural grasslands with all their characteristics [17,18]. The Agency for Payments and Intervention for Agriculture (APIA) from Romania, through payment schemes and support measures (rural development compensatory measures for commitments from PNDR 2014–2020), supports the biodiversity of HNV systems (Measure 10—Environment and Climate, Package 1—grasslands with high natural value). Applicants must follow the recommendations from the *Farmer’s Booklet on cross compliance*. Even under these conditions, large areas of HNV are constantly abandoned [19]. Evaluation methods may be ineffective. Assessing the maintenance and use of HNV grasslands for biodiversity conservation is a major challenge nowadays [20]. Grassland ecosystems (especially HNVs) need a clearer picture, which can be obtained by analyzing agricultural management indicators combined with those of biodiversity assessment [21,22]. Many researchers now recommend *Common Agricultural Policies*, evaluation by *result* (and not *by action*, as is currently the practice) throughout the European Community [23], and drawing up species lists of indicative value [24]. HNV is a topical issue at the European level and requires detailed research [25]. Even if lists of species with indicator value are developed on the basis of correlations from existing databases, they must be validated in long-term experiments [26]. Research on HNV must be carried out in long-term experiments because short-term study results may not reflect potential long-term changes [27]. The same authors recommend *that the knowledge gained from long-term fertilization experiments in ecology could be considered before applying any additional nutrients to semi-natural grasslands*. Experiments to determine the effect of organic manure fertilization on biodiversity are quite scarce, because at the European level, farmers prefer to use more slurry and less solid manure on grasslands [28]. Organic fertilization is a viable solution for maintaining and conserving biodiversity in Romania [29]. The wide biodiversity of HNV in mountainous regions was created and maintained by the application of solid manure [30,31,32].

The aim of this paper was to evaluate the long-term effect of organic fertilizers on the floristic structure, phytodiversity and productivity of an HNV system in the mountain area. The selected interval (2015–2017) presents the image of 15 years vegetation after setting up the experiment. Vegetation analysis was performed both quantitatively and qualitatively, then synthesized into three spectra: natural, ecological and agronomic in terms of value. The hypotheses assessed in the experiment were formulated in terms of the following questions: (i) What is the amount of fertilizers that determines major changes in the structure of floristic composition?; (ii) How much fertilizer does the HNV system need to maintain its conservative characteristics?; (iii) Can species with indicative value be identified both for each amount of fertilizer applied and for the type of grassland?; (iv) Which agronomic characteristics of phytocoenosis does organic fertilization influence?; (v) Which plant species develop a high biomass?

## 2. Results

### 2.1. The Influence of Organic Fertilizers on the Floristic Composition

**Figure 1:**
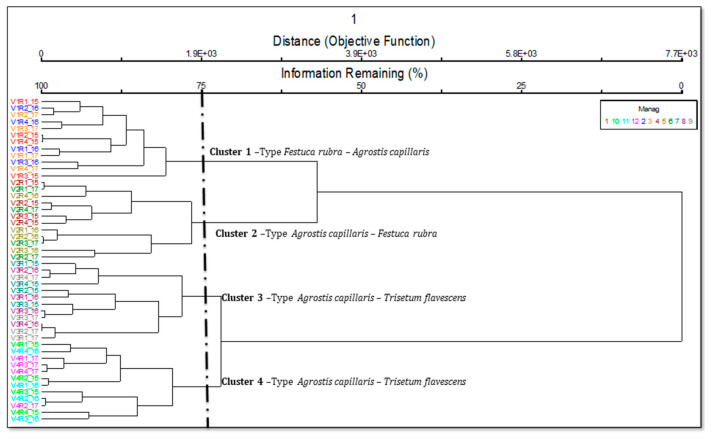
Floristic classification of the vegetation and the changes of grassland type. Legend: Manag = management; V1—control; V2—10 t ha^−1^ manure; V3—20 t ha^−1^ manure; V4—30 t ha^−1^ manure; 15, 16, 17—the three experimental years—2015, 2016, 2017; R1–4—replications.

Based on the cluster analysis, it is possible to observe the classification of the vegetation and the change of the grassland type due to the distances between their values (Figure 1). The level of cutting of the dendrogram were established based on phytosociological and ecological meaning, in order to include as much information as possible [33]. Following the analysis of the floristic composition, we considered that cutting at the value of 75 was an optimal solution, this having the highest phytosociological, ecological, and agronomic meaning. Thus, four distinct groups built by the following grasslands were identified: *Festuca rubra-Agrostis capillaris* (cluster 1), *Agrostis capillaris-Festuca rubra* (cluster 2), *Agrostis capillaris-Trisetum flavescens* cod. *Centaurea pseudophrygia* (cluster 3) and *Agrostis capillaris-Trisetum flavescens* (cluster 4). The formation of groups as a result of the input application demonstrates that fertilizer treatments produced major changes in the vegetation. There was also a clear separation in two distinct, large and diverse groups, based on treatment amount: one group represented the “low” input, corresponding to zero (V1) and 10 t ha^−1^ manure; the second group represented the “high” input and corresponded to 20 (V3) and 30 (V4) t ha^−1^ manure. Each amount of manure applied determined a different type of grassland.

**Figure 2:**
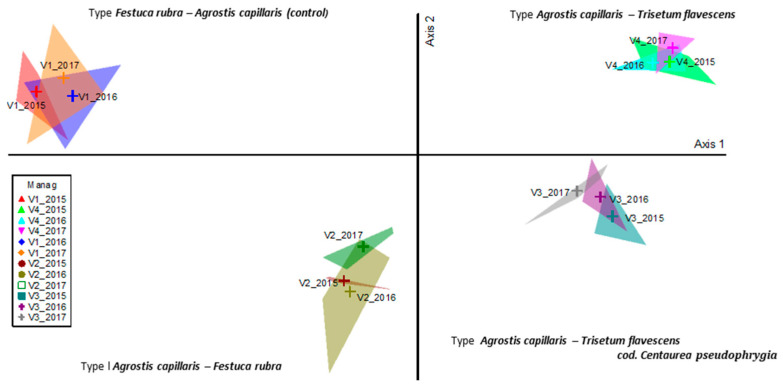
PCoA of grassland type modified by organic inputs. Legend: Manag = management; V1—control; V2—10 t ha^−1^ manure; V3—20 t ha^−1^ manure; V4—30 t ha^−1^ manure; 2015, 2016, 2017—the three experimental years.

Following the ordering with the analysis of the main coordinates (PcoA), the floristic surveys were grouped according to the similarity of floristic composition, the stationary conditions, and the applied management (Figure 2). The application of organic fertilizers (manure) caused major changes in the floristic composition. The floristic phenomenon represented a proportion of 95.5%, of which 94.0% of the observed phenomenon can be explained by the 1st ordering axis, and 0.15% by the 2nd ordering axis. Axis 1 was positively correlated with the application of quantities with 20–30 t ha^−1^ manure (*p <* 0.01), and shoed a negative correlation with the lack of fertilizers (Table 1; Figure 2). In other words, the floristic surveys determined by the treatments with 20 and 30 t ha^−1^ manure were distributed on the right side of the ordering Axis 1, and the phytocoenoses of the control can be placed on the left side of the axis. Axis 2 was positively correlated with the 10 t ha^−1^ manure treatment (*p <* 0.001), although it has a lack of biological relevance due to only 1.5% of variance being represented by this axis. PcoA sustained the phenomenon revealed by cluster analysis (Figure 1 and Figure 2), with all the phytocoenoses being distributed along Axis 1. Even Axis 2 showed a lack of importance in explanation of variance; its position makes a very clear separation between “low” and “high” input shaped phytocoenoses.

When comparing the floristic composition in the control variants between 2015 with those from 2016 and 2017, there was no significant floristic distance (V1 2015 vs. V1 2016-T = −0.230, *p <* 0.05; V1 2015 vs. V1 2017 = −0.369, *p <* 0.05; Table 2), and the floristic surveys show a high homogeneity (V1 2015 vs. V1 2016-A = −0.005). The non-existence of floristic distance within the control group indicates a weak action of the climate on the vegetal coverage in the third experimental year; these changes were small, occurred inside the phytocoenoses, and did not influence the change of grassland type during the experiment. In the case of treatment groups in the other variants, the same reduced floristic distance was found.

**Figure 3:**
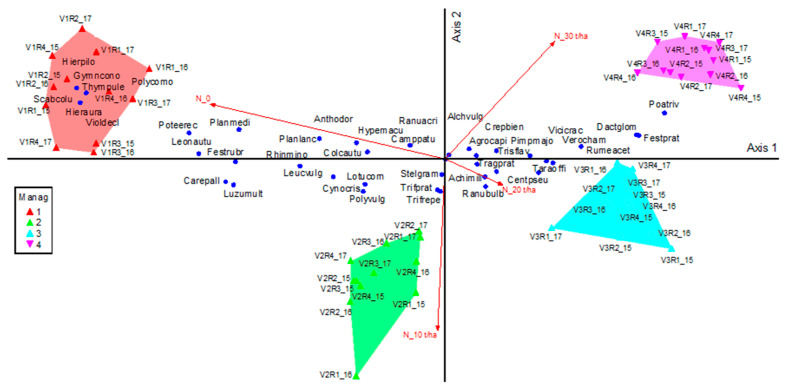
The influence of floristic composition determined by different treatments of manure. Legend: Manag = Management; V1—control; V2—10 t ha^−1^ manure; V3—20 t ha^−1^ manure; V4—30 t ha^−1^ manure. Agrocapi—*Agrostis capillaris* L.; Alchvulg—*Alchemilla vulgaris* L.; Anthodor—*Anthoxanthum odoratum* L.; Camppatu—*Campanula patula* L.; Carepall—*Carex pallescens* L.; Centpseu—*Centaurea pseudophrygia* C. A. Mey.; Colcautu—*Colchicum autumnale* L.; Crepbien—*Crepis biennis* L.; Dactglom—*Dactylis glomerata* L.; Festprat—*Festuca pratensis* Huds.; Festrubr—*Festuca rubra* L.; Gymncono—*Gymnadenia conopsea* (L.) R. Br.; Hieraura—*Hieracium aurantiacum* L.; Hierpilo—*Hieracium pilosella* L.; Hypemacu—*Hypericum maculatum* Crantz; Leonautu—*Leontodon autumnalis* L.; Leucvulg—*Leucanthemum vulgare* Lam.; Lotucorn—*Lotus corniculatus* L.; Luzumult—*Luzula multiflora* (Ehrh.) Lej.; Pimpmajo—*Pimpinella major* (L.) Huds.; Planlanc—*Plantago lanceolata* L.; Planmedi—*Plantago media* L.; Poativia—*Poa trivialis* L.; Polycomo—*Polygala comosa* Schkuhr; Polyvulg—*Polygala vulgaris* L.; Poteerec—*Potentilla erecta* (L.) Raeusch.; Ranuacri—*Ranunculus acris* L.; Ranubulb—*Ranunculus bulbosus* L.; Rhinmino—*Rhinanthus minor* L.; Rumeacet—*Rumex acetosella* L.; Scabcolu—*Scabiosa columbaria* L.; Stelgram—*Stellaria graminea* L.; Taraoffi—*Taraxacum officinale* Weber; Thympule—*Thymus pulegioides* L.; Tragprat—*Tragopogon pratensis* L.; Trifprat—*Trifolium pratense* L.; Trifrepe—*Trifolium repens* L.; Trisflav—*Trisetum flavescens* (L.) P. Beauv.; Verocham—*Veronica chamaedrys* L.; Vicicrac—*Vicia cracca* L.; Violdecl—*Viola declinata* L.

The phytocoenosis of the control treatment is represented by the *Festuca rubra*-*Agrostis capillaris* grouping, and by applying a quantity of 10 t ha^−1^ manure, it evolves into the *Agrostis capillaris*-*Festuca rubra* grouping (Figure 3; Table 3; T = −14.92; *p <* 0.001). This co-dominance between *Festuca rubra*-*Agrostis capillaris* is generally very fragile. The co-dominance of these species can be influenced even by climatic fluctuations. This situation, in the case of the 3rd experimental years, was not supported, given the weak effect of climate on the floristic composition. The application of 20 t ha^−1^ manure determines an increase in the dominance of the species *Agrostis capillaris-Trisetum flavescens* and of the species *Centaurea pseudophrygia* (T = −14.44; *p <* 0.001). In fact, the significant change in the vegetal grassland coverage occurs when applying the quantities of 20 t ha^−1^ manure. Fertilization with 30 t ha^−1^ manure determines a stronger abundance (weight) of the species *Agrostis capillaris* and *Trisetum flavescens* remains at the same level (*Agrostis capillaris*-*Trisetum flavescens* grouping).

Of all of the species presented in phytocoenosis (Table 4), 17 were negatively correlated with axis 1 and positively corelated with axis 2, which means that they were favored by the absence of fertilization or by small quantities of fertilization. Of these, the most important are: *Anthonxantum odoratum* (*p <* 0.001), *Festuca rubra* (*p <* 0.001), *Carex pallescens* (*p <* 0.001), *Gymnadenia conopsea* (*p <* 0.001), *Plantago media* (*p <* 0.001), *Polygala comosa* (*p <* 0.001). For these species, the application of 10 t ha^−1^ manure annually results in too much input of nutritive elements for them to remain at an ecological optimum. Some of the species (four) had an ecological optimum between no fertilization and the treatment with 10 t ha^−1^ manure: *Cynosurus crystatus* (*p <* 0.001), *Polygala vulgaris* (*p <* 0.001), *Scabiosa columbaria* (*p <* 0.05), *Thymus pulegioides* (*p <* 0.05).

Other species correlated with first ordering axis, which is considered the positive zone, indicating that their biomass increased with large amounts of manure as fertilization (Table 4), for instance, *Dactylis glomerata* (*p <* 0.001), *Festuca pratensis* (*p <* 0.001), *Poa trivialis* (*p <* 0.001), and *Trisetum flavescens* (*p <* 0.001). Some of the species were correlated with the 2nd axis, the negative zone, and they were favored by moderate amounts of organic fertilizers (10 t ha^−1^ manure); this group included plants such as *Trifolium pratense* (*p <* 0.001) and *Trifolium repens* (*p <* 0.001).

Plant species like Carex pallescens, Luzula multiflora, Lotus corniculatus, Crepis biennis, Gymnadenia conopsea, Leucantemum vulgare, Ranunculus bulbosus, Stellaria graminea could be favored by the application of organic fertilizers in quantities of less than 10 t ha^−1^ manure, as this had a tendency to negatively correlate with the second axis. Species Centaurea pseudophrygia could increase in biomass when quantities greater than 10 t ha^−1^ manure are applied, but at the same time it develops well when the amount of fertilizer is less than 20 t ha^−1^ manure.

The application of organic treatments has led to the extinction of some species and the emergence of others. The application of 10 t ha^−1^ manure annually caused the disappearance from the vegetation of the following species: *Gymnadenia conopsea*, *Hieracium aurantiacum*, *Hieracium pilosella*, *Thymus pulegioides*. Treatment with 20 t ha^−1^ manure resulted in the extinction of three species (*Carex pallescens*, *Luzula multiflora*, *Leontodon autumnalis*). The application of 30 t ha^−1^ manure led to the extinction of the following species: *Cynosurus cristatus*, *Polygala vulgaris*, *Potentila erecta*. The treatment with 10 t ha^−1^ manure led to the appearance of species *Dactylis glomerata* and *Festuca pratensis* and the treatment with 20 t ha^−1^ manure led to the appearance of *Poa trivialis*.

### 2.2. Indicator Species of Organic Treatments Use

Native phytocoenosis consists of 41 species, with the presence and relative abundance conditioned by the applied treatments, which is a potential indicator of plant successions (Table 5). The absence of fertilizer inputs is highlighted by a high number of species, most with very significant indicator value. Among the grasses, *Anthoxanthum odoratum* and *Festuca rubra* are perfect indicators (*p <* 0.001) of oligotrophic ecosystems (41% and 57%, respectively), a position confirmed also by *Carex pallescens* (50%) and *Luzula multiflora* (50%) within *Cyperaceae* and *Juncaceae*. From the plants category belonging to other botanical specimens, several 14 species indicate decreased interest in substrate maintenance: *Colchicum autumnale*, *Gymnadenia conopsea*, *Hieracium aurantiacum*, *Hieracium pilosella*, *Hypericum maculatum*, *Leontodon autumnalis*, *Leucanthemum vulgare*, *Plantago media*, *Polygala comosa*, *Potentilla erecta*, *Rhinanthus minor*, *Scabiosa columbaria*, *Thymus pulegioides*, *Viola declinata*, all with significant indicator values. Six of these species have a maximum indicator value for the control treatment, *Hieracium aurantiacum*, *Hieracium pilosella*, *Polygala comosa*, *Scabiosa columbaria*, *Thymus pulegioides*, *Viola declinata.* The application of 10 t ha^−1^ manure leads to the successful installation in the phytocoenosis of legumes with an indicator value of about 40% (*Lotus corniculatus*—41%, *Trifolium pratense*—38%, and *Trifolium repens*—37%), which supports the application of a low-input system to stimulate the completion of nitrogen reserves in the soil based on the symbiosis of these plants with nitrogen fixing bacteria. For this type of management, *Achillea millefolium* represents the only species with indicator value. Increasing the fertilization level to 20 t ha^−1^ manure leads to a restriction of the species strongly supported by this treatment. Only four species—*Centaurea pseudophrygia*, *Pimpinella major*, *Ranunculus bulbosus* and *Rumex acetosella*—have a significant indicator value. This aspect suggests the successive character of the phytocoenosis due to fertilization and predicts a vegetation transformation with a medium dynamism. Increasing inputs to 30 t ha^−1^ leads to a strong selection of indicator plants. The strong change in phytocoenosis indicates a rapid succession and is supported by the presence of seven species with indicator value. Compared to the control treatment, the number of species represents one-third, but in this category, there are three grasses with over 50% indicator value (*Dactylis glomerata*, *Festuca pratensis*, *Poa trivialis*), and two species with indicator value in the range of 30–35% (*Agrostis capillaris* and *Trisetum flavescens*). Only two species on the edge of the ecological niche are adapted to the large amount of fertilizer, both *Taraxacum officinale* and *Veronica chamaedrys* having a significant indicator value.

### 2.3. The Influence of Organic Fertilization on the Agro-Ecological Spectrum

Organic fertilizers act with a powerful impact on the Naturality spectrum (Table 6). The species number decreases proportionally with the increase of applied fertilizer (F = 368.80; *p* < 0.001), with significant differences for each 10 t ha^−1^ manure. Contrariwise, Shannon index shows a significant decrease only at 30 t ha^−1^ manure (V4) compared to the other three treatments. Ecological spectrum shows two similar vegetation patterns due to the fertilizer gradient applied or assessed. **U** shows a significant increase only at the highest value of applied fertilizer, compared with **N** where the trend is significantly linked to the value of applied manure dose. This parameter increases from 4.44 (Co) up to 5.26 (30 t ha^−1^). **R** is the only parameter that decreased significantly from 0 to 10 t ha^−1^ manure, followed by a slight 0.10 increase from 10 to 20 and 30 t ha^−1^ manure. The agronomic spectrum shows both increases and decreases due to applied fertilizers. The tolerance to mowing has a small increase at 10 t ha^−1^ compared to control, followed by a significant decrease at 20 and 30 t ha^−1^. The resistance of vegetation to grazing deceased drastically form control to 20 t ha^−1^, with significant differences between different treatments. The loose for this parameter was 0.42 from 0 to 10 t ha^−1^, followed by a decrease of only 0.22. This suggest that any applied manure will act as a perturbation in vegetation, but the impact is lower once the vegetation faced this change. The same trend as in grazing was visible in the decrease of tolerance to crushing. The decrease was higher from 0 to 10 t ha^−1^; after this fertilizer value, the decrease was less visible, but still significant if 10 to 20 t ha^−1^ was applied. Forage value increased significantly from 0 to 10 t ha^−1^ applied manure, but with non-significant differences between 20 to 30 t ha^−1^ treatment. The only parameter that increased significantly due to the applied manure was the dry matter yield. It started at a base of 2.01 t ha^−1^, and increased with more than 1.4 t ha^−1^ at 10 t ha^−1^ manure. The increase of yield from 10 to 20 t ha^−1^ was 0.9 t ha^−1^, and only 0.36 for more than 10 t ha^−1^ manure (V4). Based on treatment gradient, each vegetation group was characterized by a specific combination of agro-ecologic indicators. The lack of fertilizers in control treatment led to an increased number of species and a higher resistance to grazing and plants crushing.

The negative part of the control agro-ecological spectrum showed a low trophicity and a significant reduction of forage value and yield. For 10 t ha ^−1^ manure (V1), the vegetation cover was most resistant to mowing, but all the other parameters indicated a successional stage with moderate-low resistance and requirements. By increasing the fertilizer to 20 t ha^−1^ (V2), the vegetation reached a high trophicity and had a forage value. The negative aspect of this vegetation group is represented by the low values of mowing, grazing and crushing tolerance completed by a significant reduction of species number. The maximum quantity of manure (V4) applied in the experiment showed an opposite agro-ecological spectrum compared to the control. The tolerance of phytocoenosis to grazing was indirectly proportional to organic fertilization. The reduction of species is up to 30% under 30 t ha^−1^ manure. The vegetation had high values for forage quality and yield, but the vegetation is highly sensitive to mowing, grazing and crushing. An interesting case is Shannon index, with a significant decrease in this treatment. Additionally, for V4 there was a visible change of radiation sensitivity from control. This aspect sustains the anthropic-induced climax in vegetation due to the high quantity of fertilizer inputs.

The *Festuca rubra-Agrostis capillaris* grouping had an average productivity in the three experimental years of 2.01 t ha^−1^ DM. The *Agrostis capillaris*-*Festuca rubra* grouping, determined by the treatment with 10 t ha^−1^ manure, led to a DM harvest of 3.42 t ha^−1^, with a very significant difference of 1.41 t ha^−1^ DM compared to the control treatment. The *Agrostis capillaris*-*Trisetum flavescens* grouping (20 t ha^−1^ manure variant) recorded an average harvest per experimental year of 4.31 t ha^−1^ DM, with a very significant difference of 2.31 t ha^−1^ compared to the control phytocoenosis (Figure S1; Table 6). The same grouping of grassland (*Agrostis capillaris*-*Trisetum flavescens*) determined by the treatment with 30 t ha^−1^ manure, led to a harvest of 4.99 t ha^−1^ DM, with a very significant difference from the control (2.98 t ha^−1^ DM). The grassland *Agrostis capillaris*-*Trisetum flavescens* grouping (V3) brought a significant difference in yield compared to the *Agrostis capillaris*-*Festuca rubra* grouping (V2; 0.90 t ha^−1^ DM). The same grouping was determined by the treatment with 30 t ha^−1^ manure, bringing an increase of 1.5 t ha^−1^ DM compared to the phytocoenosis *Agrostis capillaris*-*Festuca rubra* (*p <* 0.05) grouping, and of 0.67 t ha^−1^ DM compared to *Agrostis capillaris*-*Trisetum flavescens* co-dominant *Centaurea pseudophrygia*.

The biomass yield was mainly developed at the expansion of the following species: *Agrostis capillaris*, *Trisetum flavescens*, *Centaurea pseudophrygia*, *Taraxacum officinale*, *Veronica chamaedrys*. Species *Agrostis capillaris* (Figure S2) was moderately influenced by the applied treatments and had the highest share in the treatments with 20 and 30 t ha^−1^ manure *r* = 0.692 (Figure S6). The species increased its share from 13.8% coverage (control) to 20.3% when applying 30 t ha^−1^ manure. Species *Trisetum flavescens* (Figure S3) was strongly influenced by the applied treatments and had the highest share in the treatments with 20 and 30 t ha^−1^ manure *r* = 0.883, and increased its share from 5.6% coverage (control) to 9.9% (V2—10 t ha^−1^ manure), reaching up to 15.8% in the most intensively fertilized version (30 t ha^−1^ manure). Species *Centaurea pseudophrygia* (Figure S4) was moderately influenced by the applied treatments and had the highest share with the treatments with 20 t ha^−1^ manure *r* = 0.507. This species increased its share from 5.5% coverage (control) to 11.75% (20 t ha^−1^ manure), after which it registered a decrease in the dominance of the vegetal coverage to 7.5% with treatment with 30 t ha^−1^ manure. *Taraxacum officinale* (Figure S5) was moderately influenced by the treatments applied and had the highest share with treatments with 10, 20 and 30 t ha^−1^ manure *r* = 0.702. The dominance of the species increased from 1% coverage (control) to 5.1% when applying the treatment with 10 t ha^−1^ manure, 4.4% with the treatment with 20 t ha^−1^ manure, and reaching 5.6% (30 t ha^−1^ manure). *Veronica chamaedrys* (Figure S6) was moderately to strongly influenced by the applied treatments and had the highest share in the treatments with 30 t ha^−1^ manure *r* = 0.873. The species increased its share from 1.2% coverage (control) to 3.2% for the treatment with 10 t ha^−1^ manure, 6.3% for the fertilized treatments with 20 t ha^−1^ manure, reaching a coverage of 8.1% in the vegetal expanse for the most intensive fertilized version (30 t ha^−1^ manure). The application of organic fertilizer led to an improvement in feed quality. If the control phytocoenosis (*Festuca rubra-Agrostis capillaris*) falls into class V (grassland predominated by species with an average forage value, which supports 0.81–1.00 UVM ha^−1^), the average category, and after fertilization with manure there was a classification in class VII quality, the good category (V4—30 t ha^−1^ manure; grouping *Agrostis capillaris*-*Trisetum flavescens*), a grassland was dominated by a species with a good forage value, which supports 1.21–1.50 UVM ha^−1^. This increase in quality can be explained by the installation of new types of grassland due to the increase in system intensity. Due to the codominance of the species *Centaurea pseudophrygia*, the *Agrostis capillaris*-*Trisetum flavescens* grouping falls into class VI (middle category).

## 3. Discussion

The application of organic fertilization on *Festuca rubra* grassland with a high biodiversity for a long time determined a clear classification of phytocoenoses. The management intensity of the grasslands is what determines a clear classification of the surveys according to the similarity of floristic composition [29,33]. Following the treatments, some species settle as dominant or co-dominant. The application of different fertilizer doses contributed to major changes in the coverage, signaling that organic treatments determine the dominance of some species and the extinction of others [34]. Species *Agrostis capillaris* increases its share as the system intensifies, being present in all phytocoenoses [35]. Under conditions of organic fertilization, most often, the species *Agrostis capillaris* is present as a co-dominant [36]. Species *Festuca rubra* reduces its weight as the management intensifies with organic inputs, being co-dominant from the application of 20 t ha^−1^ manure. Dominance of the species *Festuca rubra* is a cumulative result of the application of organic fertilizers (≤10 t ha^−1^) and climatic factors [37]. The application of the quantities of 20 t ha^−1^ manure determined the installation of the *Agrostis capillaris-Trisetum flavescens* in the grassland. This grassland is also identified in the central area of the Apuseni Mountains on fertile land, flat and fertilized with large amounts of manure [38]. Organic fertilization of a combination *Agrostis capillaris-Festuca rubra* grassland, leads to an increased participation of the species *Trisetum flavescens* [39]. Some plant species are favored by the fertilization absence or by small quantities (10 t ha^−1^ manure) of fertilization. The results obtained by us are confirmed by other studies who identify several species with a high conservation value (*Gymnadenia conopsea*) in extensive used grasslands [40]. The results are also confirmed [41], and the species founded in the oligotrophic and oligomesotrophic habitats of *Arnica montana* (*Festuca rubra*, *Carex pallescens*, *Gymnadenia conopsea*, *Plantago media* etc.) have been identified. The species *Polygala comosa* and *Scabiosa columbaria* have been identified in extensive grasslands management in Poland [42] and in oligotrophic grasslands from Italy [43]. *Scabiosa columbaria* plants were maintained by small amounts of fertilizers inputs and removed by intensive fertilization [44], which confirms the results obtained in this study [45,46]. Fertilization with 10 t ha^−1^ manure favored the species *Thymus pulegioides*, also confirmed in other ecosystems [47]. Species *Trifolium pratense* and *Trifolium repens* are favored by the application of 10 t ha^−1^ manure, a situation confirmed by many other studies [46,48,49]. Grass plant species (*Dactylis glomerata*, *Festuca pratensis*, *Poa trivialis*, *Trisetum flavescens*) prefer quantities of 20–30 t ha^−1^ organic fertilizer, some of them even becoming dominant. Ref. [49] showed that long-term organic fertilization with goat manure (applied pure or in combination with NPK) favored these grass species. The application of small or moderate amounts of organic input (10 t ha^−1^ manure once every 2–3 years or 5 t ha^−1^ annually) was able to stimulate some specific species with high importance for the biodiversity of grasslands systems (*Carex pallescens*, *Luzula multiflora*, *Lotus corniculatus*, *Crepis biennis*, *Gymnadenia conopsea*, *Leucantemum vulgare*, *Ranunculus bulbosus*, *Stellaria graminea*). These species have been identified in grasslands used traditionally, extensively or semi-extensively by grazing or mowing, and most often fertilized with small or moderate amounts of organic and sometimes even mineral fertilizer [46,50,51,52,53,54]. Further intensification of the grassland system (20–30 t ha^−1^ manure) leads to an increase in the covering area of the species *Centaurea pseudophrygia*, which is of neither conservational nor agronomic importance [45,46,55]. Annual fertilization with 10 t ha^−1^ manure causes a restriction of biodiversity and the extinction of some species (*Gymnadenia conopsea*, *Hieracium aurantiacum*, *Hieracium pilosella*, *Thymus pulegioides*). *Gymnadenia conopsea* and *Thymus pulegioides* are identified in species-rich, nutrient-poor, extensively managed grasslands [56,57,58,59]. *Hiaracium pillosela* grows very well on poor soils, where grasslands have gaps and become invasive in different regions of the world [60,61]. The intensification of the grassland management determines the disappearance of the species from phytocoenosis [62]. *Hieracium aurantiacum* grows on weak to moderately nitrophilous substrates [56] and occurs invasively in Australia [63]. The treatment with 20 t ha^−1^ manure further determines the biodiversity restriction (*Carex pallescens*, *Luzula multiflora*, *Leontodon autumnalis*). *Luzula multiflora* is present in oligotrophic or mesotrophic grassland, and K-based fertilization favors it [64]. *Leontodon autumnalis* is disadvantaged by this fertilizer amount, because it is a weakly competitive species in the lush conditions of vegetation clearing determined by the fertilizer application. Frequent mowing (2–4 times a year) and moderate fertilization favor the species [65]. Fertilization with quantities over 100 N kg ha^−1^ favors the species [66], while organic fertilization with 20 t ha^−1^ manure applied every 1/2 years favors the species [47]. Treatment with 30 t ha^−1^ manure causes the disappearance of three species from the vegetation cover: *Cynosurus cristatus*, *Polygala vulgaris*, *Potentila erecta*. *Cynosurus cristatus* has been identified in the *Festuca rubra-Agrostis capillaris* grasslands [51], with this grassland being maintained by organic fertilization with quantities between 10 and 20 t ha^−1^ manure [38]. Under the conditions of application of 30 t ha^−1^ manure, the species most likely disappeared due to the more aggressiveness and competitive capacity of the dominant species. *Polygala vulgaris* and *Potentila erecta* are typical species for low-fertility habitats [62]. In our experiment, they withstood the competition of other species up to the treatment with 20 t ha^−1^ manure.

The grassland systems (HNV) in South-Eastern Europe and the Carpathian Mountains have a wide biodiversity range [16,67,68]. HNV grasslands of *Festuca rubra*-*Agrostis capillaris* from the Apuseni Mountains have a high biodiversity and are traditionally managed by a mixed system (mowing + extensive autumn grazing) and fertilized with small amounts of manure [38,46,69]. Species *Festuca rubra* shows indicative value for HNV grasslands in mountain areas, unfertilized or fertilized with low or moderate amounts of fertilizer [70]. The same indicator value for HNV systems is presented by the species *Athoxanthum odoratum* [17,71]. *Carex pallescens* and *Luzula multiflora* have a good indicator value for HNV systems [38,46,72]. *Gymnadenia conopsea*, *Potentilla erecta* and *Viola declinata* are identified by the same authors as species with indicative value for HNV systems. *Colchicum autumnale* is a species with indicative value for HNV systems, but it is toxic to animals and nature conservation regulations have to be optimized in regard to nature conservation goals [73]. Perhaps its use as a medicinal plant would reduce the size of its population [74]. *Leucanthemum vulgare*, *Hieracium aurantiacum* and *Hieracium pilosella* they are species with indicator values for HNV in the Apuseni Mountains and in Romania [75,76], but in other regions of the world they are invasive, and they must be monitored and kept under control [77]. *Hypericum maculatum* has indicative value for HNV, but is present in irregularly managed grasslands and sometimes even correlates with biodiversity loss [78]. Additionally, the species is a high potential indicator for landscapes with low yield potential [79]. *Polygala comosa* has indicative value for HNV, but it is also indicative for mid-successional grasslands [80]. Species *Scabiosa columbaria* it is indicative of calcareous substrates and ancient grasslands [81]. *Viola declinata* is a widespread species in the *Crex Crex* grasslands in Romania and is a Carpathian endemic species [82]. *Thymus pulegioides* has indicative value for calcareous grasslands in Central Europe [83]. It sometimes occurs in rare or endangered phytocoenoses [84]. *Rhinanthus minor* is an indicator species for HNV systems, and can be optimally spread only in grasslands with a harvest level of up to 5 t ha^−1^ SU [85]. *Plantago media* is a very common species in traditionally managed HNV mountain grasslands and fertilized with small amounts of manure [38,45,46]. The species is in the same situation as *Leontodon autumnalis*, which is not affected by mowing or grazing [86]. Some *Fabaceae* (*Lotus corniculatus*, *Trifolium pratense* and *Trifolium repens*) are species with indicative value for organic fertilization with 10 t ha^−1^ manure. Their ecological optimum is found on oligo-mesotrophic (*Lotus corniculatus*) and mesotrophic soils [87,88]. *Achillea millefolium* is indicative of the same treatment, preferring mesotrophic substrates. This treatment causes a change in the co-dominance of the grassland, but it is also part of the HNV, because even if there is a slight reduction in specific richness (number of species), at the same time there is a slight increase in the Shannon index. This situation results in the phytocoenosis being more balanced. Some species from other botanical families (presented as Forbs in grasslands) (*Centaurea pseudophrygia*, *Pimpinella major*, *Rumex acetosella*, *Ranunculus bulbosus*) show indicative value for fertilization with 20 t ha^−1^ manure and implicitly for the grassland *Agrostis capillaris-Trisetum flavescens* cod. *Centaurea pseudophrygia*. Our results are contradictory to those obtained in Western Europe in terms of the species *Centaurea pseudophrygia.* It is described as an oligo-mesotrophic species [88,89], which would imply an annual fertilization with a maximum of 10 t ha^−1^ manure. This situation shows that the species has a different behavior in the region of Southeast Europe. A similar situation has been recorded for the species *Rumex acetosella* and *Ranunculus bulbosus* [46]. The question is whether this grassland can still be considered HNV? In the literature, there is a lack of observations regarding the association between *Agrostis capillaris* and *Trisetum flavescens*. This grassland was installed in our experience in four replicates and was identified in 37 sites in the central area of the Apuseni Mountains [38]. This grassland has a high biodiversity of 31 species in 10 m^2^ and oligomesotrophic and mesotrophic indicator species, which gives it a high natural value. *Trisetum flavescens* grasslands are considered in Western Europe as phytocoenoses spread on rich soils, but with a high specific richness of 35 species to 25 m^2^ [89]. In Romania, *Trisetum flavescens* grasslands are considered HNV [90]. Fertilization with 30 t ha^−1^ manure is indicated by species *Poaceae* (*Dactylis glomerata*, *Festuca pratensis* and *Poa trivialis*). These possess indicative value for *Agrostis capillaris-Trisetum flavescens* grassland, where *Centaurea pseudophrygia* has lost its co-dominance. These indicator species are supported by intensive grassland management, and their presence in phytocoenoses shows reduced biodiversity [91,92]. The grassland of *Agrostis capillaris-Trisetum flavescens* determined by the application of 30 t ha^−1^ manure cannot be included in the conditions of HNV, given the level of inputs and the preferences of indicator species to trophicity and management intensity, even if the specific richness is not very low.

The reduction of biodiversity as the system intensifies has been demonstrated by many studies in several natural habitats and agro-ecosystems [20,93,94]. The effect of organic manure fertilization on biodiversity is very little studied and therefore there have not been much scientific data published. Fertilization with 30 t ha^−1^ manure applied annually cannot maintain the biodiversity of HNV, because it caused the extinction of eight species. In addition, it caused an increase in DM harvest of only 0.46 t ha^−1^ and a minimum feed value compared to the 20 t ha^−1^ manure treatment, and is not economically justified. Maybe fertilizing with 20 t ha^−1^ organic fertilizer could be a maintenance option, but it also caused the extinction of another six species. This results in an extra harvest of almost 1 t ha^−1^ DM and can be attractive to farmers. The application of this amount of manure once every 2 years would be more efficient, both from an ecological and agronomic point of view, but this aspect still needs to be studied. Fertilization with 10 t ha^−1^ manure produces a minimal effect on biodiversity and brings an increase in harvest biomass of 1.4 t ha^−1^ DM [45].

The intensification of grassland systems has repercussions on the agro-ecological compositional spectrum [95,96]. Decreased tolerance of phytocoenoses to grazing and crushing as the mowing system intensifies is a usual aspect, as the tolerant to defoliation species are getting installed. Phytocoenosis representative of the control is medium tolerant to grazing (**P** = 5.29), where grazing only partially consumes the plants and is reduced to moderately tolerant to treatment with 30 t ha^-1^ manure (**P** = 4.59; grazing being a free-extensive system). This situation is explained by the new species appearance (as *Trisetum flavescens*) medium-sized and tall, and have a lower tolerance to grazing compared to control phytocoenosis species (an example would be *Festuca rubra*). Based on the results obtained, the crushing tolerance is indirect proportional to the organic fertilization, finding a decrease in the plants fall-down tolerance from the phytocoenoses. If in the control phytocoenosis, the tolerance to crushing is 4.73; after organic fertilization and the installation of new grasslands, this is reduced to a value of 4.39 cows ha^−1^. There is no change in the agronomic category, with all being moderately tolerant to crushing, which according to the literature results in crushing taking place 3–7 times during a growing season.

Control phytocoenosis productivity is comparable to *Agrostis capillaris* grassland, with average productivity, described by many specialists during research on grassland ecosystems [97]. The biomass crop of the control increases as the amount of organic fertilizers increases; a situation found by many researchers [98]. The biomass harvest developed mainly due to there being only a few species from the phytocoenosis. Ref. [99] showed that out of the multitude of species in a phytocoenosis, only some are produced by biomass, their contribution being between 80–90%. Species *Agrostis capillaris* and *Trisetum flavescens* favored intensification contributes greatly to the formation of the productivity level [100].

## 4. Materials and Methods

**Figure 4:**
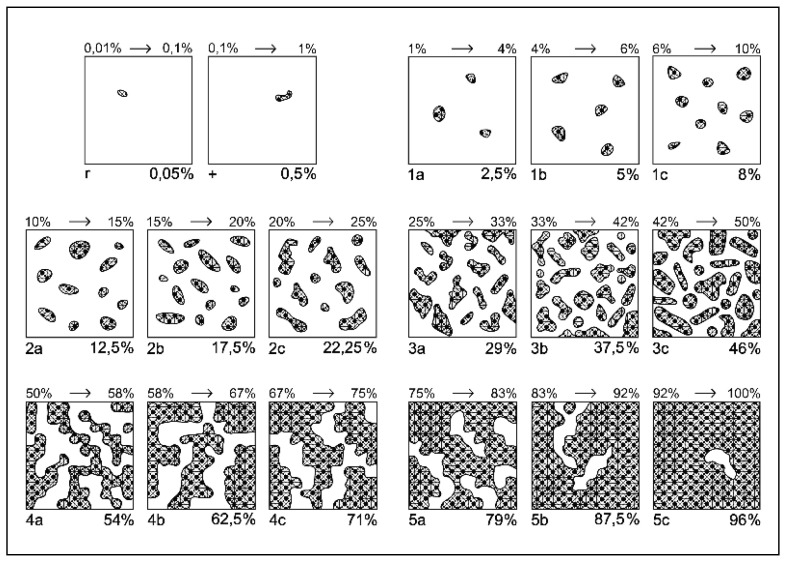
Modified Braun-Blanquét scale for grasslands, based on species coverage (after [102]). Legend: 1 to 5 indicates the class of coverage; a, b, c indicates the sub-note of each class.

The research was conducted in Ghețari area, Gârda de Sus village, Alba County, Apuseni Mountains, at 1130 m altitude on a mountain grassland. The experiment was a part of long-term research which was set up in 2001 on a red preluvosol soil, characterized by poor nutrient supply [101]. The experimental design was organized in a complete randomized blocks method, with four replications for each treatment. The experiment highlights the effect of four organic treatments gradient which consist of: T1—control, T2—10 t ha^−1^ manure, T3—20 t ha^−1^ manure, T4—30 t ha^−1^ manure over native vegetation. The technological inputs administration took place annually in early spring. The organic fertilizer came from cattle and horses having the following elements content (kg t^−1^ dry matter): 0.40 N, 0.39 P and 0.45 K. The floristic studies were performed according to a modified Braun-Blanquét method (Figure 4, Table 7), with smaller intervals adapted for a better and more realistic vegetation assessment [102]. We used data from an interval of three experimental years (2015, 2016, 2017), which highlights the cumulative effect of organic and mineral inputs after 15–17 years from the establishment of the experiment.

Vegetation was quantitatively analyzed with ANOVA and LSD test, based on the method from the “agricolae” package (de Mendiburu, 2020) in RStudio, version 1.2.5033 [103,104]. Vegetation traits were calculated as three spectra: **Naturality**—*Number of species* (Spp. no.) *Shannon Index* (Shannon); **Ecologic**—*Trophicity* (N); *Soil reaction* (R); *Humidity* (U) and **Agronomic**—*Mowing* (C); *Grazing* (P); *Crushing* (S); *Forage value* (VF); *Yield* (Y). For complete and unified analysis of the three spectra, we use the term *Agro-ecological spectrum*. Floristic data processing was performed with PC-ORD, version 7, which uses the multivariate analysis of botanical data [105]. The ordination of floristic composition was performed according to the applied fertilization and was done using the Principal Coordinates Analysis (PCoA) method [106]. PcoA is best suited to the analysis of environmental matrices and biotic data, particularly where the variables are different data types, including ranked and multistate data [107]. Principal coordinates analysis (PCoA) operates by finding a final solution in which the measured distances among sample units (on the ordination axes) correspond as well as possible to the measured distances observed in the original data; this is a form of metric multidimensional scaling [108]. All distances are calculated based on Sorenson (Bray-Curtis) formula.

## 5. Conclusions

The application of organic fertilizers was felt in each of the three treatments, installing specific phytocoenoses. In the treatment with 10 t ha^−1^ manure, compared to the control, there were no significant changes, but only a change of dominance; instead from the treatment application with 20 t ha^−1^ manure, there was an imbalance in phytocoenosis, installing an *Agrostis capillaris*-*Trisetum flavescens* cod. *Centaurea pseudophrygia* type of grassland. Further intensification of the system (30 t ha^−1^ manure) led to the installation of *Agrostis capillaris*-*Trisetum flavescens*. Each fertilization treatment determined species with indicative value both for the intensification level and for the phytocoenoses of specific meadows. Fertilization of *Festuca rubra*-*Agrostis capillaris* HNV grassland maintained HNV conditions until application of 20 t ha^−1^ organic fertilizer. The loss of the codominant species due to the intensification and further of the phytocoenosis led to the loss of HNV quality. The development of species with an indicator value for HNV systems could be very useful in their identification and management. Some species with indicative value have different preferences for trophicity in the Apuseni Mountains compared to Western European conditions. Perhaps an adaptation of species preferences to ecological factors is required, for the specific conditions from South-Eastern Europe, in the context of seasonal experiments in conjunction with the existing literature database. The application of 10 t ha^−1^ manure ensures an increase in yield and has a small influence on diversity and could be a real possibility of maintaining and sustainable use of HNV.

## Figures and Tables

**Table 1:** Correlation of experimental factors with the ordination axis.

Experimental Factors	Axis 1		Axis 2	
	*r*	Significance	*r*	Significance
Control	0.867	-	−0.419	-
10 t ha^−1^ manure	0.157	ns	0.741	***
20 t ha^−1^ manure	−0.430	*	0.291	ns
30t ha^−1^ manure	−0.594	**	−0.613	***
Axis importance		94.0%		1.5%

Note: *r*—correlation coefficient between ordination distances and original distances in *n*-dimensional space; Significance: *p <* 0.001 ***; *p <* 0.01 **; *p <* 0.05 *; ns—not significant.

**Table 2:** Three-year comparison of floristic composition changes due to applied treatments (MRPP).

Treatments	T	A	*p*.val
V1_2015 vs. V1_2016	−0.230	0.005	0.406
V1_2015 vs. V1_2017	−0.369	0.009	0.347
V2_2015 vs. V2_2016	−2.238	0.078	0.022 *
V2_2015 vs. V2_2017	−0.772	0.027	0.213
V3_2015 vs. V3_2016	1.228	−0.034	0.913
V3_2015 vs. V3_2017	0.424	−0.012	0.641
V4_2015 vs. V4_2016	1.555	−0.051	0.946
V4_2015 vs. V4_2017	−0.702	0.038	0.035 *

Note: V1—control; V2—10 t ha^−1^ manure; V3—20 t ha^−1^ manure; V4—30 t ha^−1^ manure; T—T test, A—group homogeneity; Significance: *p <* 0.05 *; ns—not significant.

**Table 3:** Comparison of floristic composition changes due to applied treatments (MRPP).

Treatments	T	A	*p*.val
V1 vs. V2	−14.92	0.295	*p* < 0.001
V1 vs. V3	−15.62	0.487	*p* < 0.001
V1 vs. V4	−15.69	0.529	*p* < 0.001
V2 vs. V3	−14.44	0.260	*p* < 0.001
V2 vs. V4	−15.29	0.364	*p* < 0.001
V3 vs. V4	−11.64	0.136	*p* < 0.001

Note: T—T test, A—group homogeneity; Significance: *p <* 0.001 ***; *p <* 0.01 **; *p <* 0.05 *; ns—not significant.

**Table 4:** Plant species correlation with the ordination axis in long term fertilized grassland.

Species	Axis 1				Axis 2			
	*r*	*r-sq*	*tau*	*Signif.*	*r*	*r-sq*	*tau*	*Signif.*
*Agrostis capillaris* L.	0.692	0.479	−0.599	***	0.200	0.040	−0.184	ns
*Anthoxanthum odoratum* L.	−0.640	0.410	0.418	***	−0.312	0.097	−0.203	ns
*Cynosurus cristatus* L.	−0.594	0.353	0.605	**	−0.613	0.376	0.521	***
*Dactylis glomerata* L.	0.797	0.635	−0.749	***	0.256	0.065	−0.098	ns
*Festuca pratensis* Huds.	0.854	0.730	−0.804	***	0.286	0.082	−0.151	ns
*Festuca rubra* L.	−0.984	0.968	0.844	***	0.027	0.001	0.198	ns
*Poa trivialis* L.	0.595	0.354	−0.713	**	0.326	0.106	−0.289	ns
*Trisetum flavescens* (L.) P. Beauv.	0.883	0.780	−0.770	***	0.092	0.009	−0.108	ns
*Carex pallescens* L.	−0.887	0.786	0.715	***	−0.279	0.078	0.258	ns
*Luzula multiflora* (Ehrh.) Lej.	−0.882	0.779	0.709	***	−0.233	0.054	0.217	ns
*Lotus corniculatus* L.	−0.380	0.144	0.309	*	−0.305	0.093	0.241	ns
*Trifolium pratense* L.	−0.066	0.004	0.206	ns	−0.666	0.444	0.549	***
*Trifolium repens* L.	−0.049	0.002	0.166	ns	−0.806	0.650	0.671	***
*Vicia cracca* L.	0.635	0.403	−0.532	***	0.031	0.001	0.050	ns
*Achillea millefolium* L.	0.283	0.080	−0.165	ns	−0.509	0.260	0.360	**
*Alchemilla vulgaris* L.	0.055	0.003	−0.019	ns	0.162	0.026	−0.128	ns
*Centaurea pseudophrygia* C. A. Mey.	0.507	0.257	−0.306	**	−0.583	0.340	0.398	**
*Colchicum autumnale* L.	−0.613	0.375	0.470	***	0.148	0.022	−0.078	ns
*Crepis biennis* L.	0.110	0.012	−0.077	ns	0.129	0.017	−0.110	ns
*Gymnadenia conopsea* (L.) R. Br.	−0.637	0.406	0.469	***	0.320	0.103	−0.255	ns
*Hieracium aurantiacum* L.	−0.867	0.751	0.619	***	0.419	0.176	−0.304	*
*Hieracium pilosella* L.	−0.867	0.751	0.619	***	0.419	0.176	−0.304	*
*Hypericum maculatum* Crantz	−0.274	0.075	0.195	ns	0.280	0.079	−0.215	ns
*Leontodon autumnalis* L.	−0.669	0.447	0.710	***	0.039	0.002	0.208	ns
*Leucanthemum vulgare* Lam.	−0.720	0.518	0.646	***	−0.292	0.085	0.276	ns
*Pimpinella major* (L.) Huds.	0.581	0.337	−0.479	**	0.248	0.061	−0.210	ns
*Plantago lanceolata* L.	−0.601	0.361	0.502	***	0.255	0.065	−0.162	ns
*Plantago media* L.	−0.649	0.421	0.454	***	0.246	0.060	−0.213	ns
*Polygala comosa* Schkuhr	−0.867	0.751	0.619	***	0.304	0.176	−0.304	ns
*Polygala vulgaris* L.	−0.594	0.353	0.605	**	−0.613	0.376	0.521	***
*Potentilla erecta* (L.) Raeusch.	−0.824	0.680	0.766	***	0.218	0.047	0.099	ns
*Ranunculus bulbosus* L.	0.209	0.044	−0.108	ns	−0.129	0.017	0.090	ns
*Rhinanthus minor* L.	−0.720	0.519	0.533	***	−0.086	0.007	0.083	ns
*Rumex acetosella* L.	0.718	0.515	−0.535	***	0.059	0.003	0.010	ns
*Scabiosa columbaria* L.	−0.867	0.751	0.619	***	0.419	0.176	−0.304	*
*Stellaria graminea* L.	−0.023	0.001	0.025	ns	−0.240	0.057	0.205	ns
*Taraxacum officinale* Weber	0.702	0.493	−0.554	***	−0.258	0.066	0.194	ns
*Thymus pulegioides* L.	−0.867	0.751	0.619	***	0.419	0.176	−0.304	*
*Tragopogon pratensis* L.	0.437	0.191	−0.296	*	−0.177	0.031	0.153	ns
*Veronica chamaedrys* L.	0.873	0.761	−0.772	***	0.211	0.044	−0.174	ns
*Viola declinata* L.	−0.645	0.416	0.605	***	0.262	0.068	−0.279	ns

Note: *r*—Pearson correlation coefficient; *r-sq*—determination coefficient; *tau*— rank (Kendall’s tau) relationships between the ordination scores and the individual variables; Significance: *p <* 0.001 ***; *p <* 0.01 **; *p <* 0.05 *; ns—not significant.

**Table 5:** Indicator value of species related to applied treatments.

Species	V1	V2	V3	V4	Group Indval	Signif.
*Agrostis capillaris* L.	21	24	26	30	4	*p* < 00.1
*Anthoxanthum odoratum* L.	41	26	10	23	1	*p* < 00.1
*Cynosurus cristatus* L.	33	33	33	0	1	0.064
*Dactylis glomerata* L.	0	8	41	51	4	0.001
*Festuca pratensis* Huds.	0	11	37	53	4	*p* < 00.1
*Festuca rubra* L.	57	33	8	1	1	*p* < 00.1
*Poa trivialis* L.	0	0	38	63	4	*p* < 00.1
*Trisetum flavescens* (L.) P. Beauv.	12	22	31	35	4	0.001
*Carex pallescens* L.	50	50	0	0	1	*p* < 00.1
*Luzula multiflora* (Ehrh.) Lej.	52	44	0	0	1	*p* < 00.1
*Lotus corniculatus* L.	32	41	14	14	2	0.034
*Trifolium pratense* L.	19	38	26	17	2	*p* < 00.1
*Trifolium repens* L.	19	37	28	16	2	*p* < 00.1
*Vicia cracca* L.	7	28	23	41	4	0.001
*Achillea millefolium* L.	12	37	30	21	2	0.009
*Alchemilla vulgaris* L.	25	24	25	27	4	0.747
*Centaurea pseudophrygia* C. A. Mey.	16	28	34	22	3	*p* < 00.1
*Colchium autumnale* L.	38	25	19	18	1	*p* < 00.1
*Crepis biennis* L.	25	15	35	25	3	0.275
*Gymnadenia conopsea* (L.) R. Br.	58	0	0	0	1	0.001
*Hieracium aurantiacum* L.	100	0	0	0	1	*p* < 00.1
*Hieracium pilosella* L.	100	0	0	0	1	*p* < 00.1
*Hypericum maculatum* Crantz	34	21	21	25	1	0.040
*Leontodon autumnalis* L.	63	38	0	0	1	*p* < 00.1
*Leucanthemum vulgare* Lam.	38	36	15	11	1	0.003
*Pimpinella major* (L.) Huds.	19	18	31	31	3	0.026
*Plantago lanceolata* L.	49	18	18	14	1	*p* < 00.1
*Plantago media* L.	67	12	8	12	1	*p* < 00.1
*Polygala comosa* Schkuhr	100	0	0	0	1	*p* < 00.1
*Polygala vulgaris* L.	33	33	33	0	1	0.064
*Potentilla erecta* (L.) Raeusch.	76	12	12	0	1	*p* < 00.1
*Ranunculus bulbosus* L.	18	18	46	18	3	0.013
*Rhinanthus minor* L.	43	43	0	5	1	0.001
*Rumex acetosella* L.	8	21	39	32	3	0.001
*Scabiosa columbaria* L.	100	0	0	0	1	*p* < 00.1
*Stellaria graminea* L.	21	36	21	21	2	0.227
*Taraxacum officinale* Weber	6	32	27	35	4	0.010
*Thymus pulegioides* L.	100	0	0	0	1	*p* < 00.1
*Tragopogon pratensis* L.	12	27	27	27	2	0.895
*Veronica chamaedrys* L.	6	17	33	43	4	*p* < 00.1
*Viola declinata* L.	100	0	0	0	1	*p* < 00.1

Note: V1—control; V2—10 t ha^−1^ manure; V3—20 t ha^−1^ manure; V4—30 t ha^−1^ manure; Significance: *p <* 0.001 ***; *p <* 0.01 **; *p <* 0.05 *; ns—not significant.

**Table 6:** The influence of organic fertilizer gradient over agro-ecologic spectra.

**Var**	**Spp. No.**	**Shannon**	**U**	**R**	**N**
V1	37.92 ± 1.88 a	2.91 ± 0.08 a	5.05 ± 0.06 b	5.81 ± 0.19 a	4.44 ± 0.14 c
V2	34.67 ± 0.49 b	2.94 ± 0.05 a	5.00 ± 0.07 b	5.56 ± 0.13 b	5.01 ± 0.11 b
V3	31.33 ± 0.49 c	2.89 ± 0.08 a	5.04 ± 0.08 b	5.67 ± 0.15 ab	5.17 ± 0.1 a
V4	29.67 ± 0.49 d	2.72 ± 0.09 b	5.11 ± 0.04 a	5.76 ± 0.2 a	5.26 ± 0.11 a
*F test*	386.80	30.91	5.98	0.04	139.53
*p.val*	*p* < 0.001	*p* < 0.001	0.018	0.842	*p* < 0.001
**Var**	**C**	**P**	**S**	**VF**	**Yield**
V1	6.44 ± 0.1 ab	5.29 ± 0.13 a	4.73 ± 0.12 a	5.39 ± 0.15 c	2.01 ± 0.37 d
V2	6.5 ± 0.11 a	4.87 ± 0.19 b	4.53 ± 0.12 b	5.82 ± 0.11 b	3.42 ± 0.27 c
V3	6.37 ± 0.07 bc	4.65 ± 0.12 c	4.42 ± 0.07 c	5.91 ± 0.17 ab	4.31 ± 0.49 b
V4	6.33 ± 0.07 c	4.62 ± 0.12 c	4.38 ± 0.06 c	6.03 ± 0.11 a	4.77 ± 0.34 a
*F test*	13.01	98.79	78.95	91.91	261.12
*p.val*	0.001	*p* < 0.001	*p* < 0.001	*p* < 0.001	*p* < 0.001

Note: V1—control; V2—10 t ha^−1^ manure; V3—20 t ha^−1^ manure; V4—30 t ha^−1^ manure; Means ± s.e. followed by different letters indicate differences at *p* < 0.05 according to LSD test; Significance: *p* *<* 0.001 ***; *p* *<* 0.01 **; *p* *<* 0.05 *; ns—not significant. Spp. No—species number; Shannon—Shannon Index; U—Humidity; R—soil reaction; N—Trophicity; C—mowing; P—grazing; S—Crushing; VF—forage value; Yield—dry matter yield ha^−1^.

**Table 7:** Modified Braun-Blanquét scale for assessing the abundance-dominance of plant species, based on classes and sub-classes (after [102]).

Class	Coverage Interaval (%)	Class Central Value (%)	Sub-Note	Sub-Interval (%)	Central-Adjusted Value of Sub-Interval (%)
5	75–100	87.5	5 c	92–100	96
			5 b	83–92	87.5
			5 a	75–83	79
4	50–75	62.5	4 c	67–75	71
			4 b	58–67	62.5
			4 a	50–58	54
3	25–50	37.5	3 c	42–50	46
			3 b	33–42	37.5
			3 a	25–33	29
2	10–25	17.5	2 c	20–25	22.25
			2 b	15–20	17.5
			2 a	10–15	12.5
1	1–10	5	1 c	6–10	8
			1 b	4–6	5
			1 a	1–4	2.5
+	0.1–1	0.5	-	-	0.5
r	0.01–0.1	0.05	-	-	0.05

a, b, c indicates the sub-note of each class.

## Data Availability

Data is contained within the article or Supplementary Materials.

## Supplementary Materials

The following are available online at https://www.mdpi.com/article/10.3390/plants10040739/s1, Figure S1. The influence of organic fertilizer on the dry matter (DM) yield; Figure S2. The influence of *Agrostis capillaris* species on the dry matter (DM) yield; Figure S3. The influence of *Trisetum flavescens* species on dry matter (DM) yield; Figure S4. The influence of *Centaurea pseudophrygia* species on the dry matter (DM) yield; Figure S5. The influence of *Taraxacum officinale* species on the dry matter (DM) yield; Figure S6. The influence of *Veronica chamaedrys* species on the dry matter (DM) yield.

## References

[1] Buchmann N., Fuchs K., Feigenwinter I., Gilgen A.K. (2019). Multifunctionality of Permanent Grasslands: Ecosystem Services and Resilience to Climate Change. Res. Collect..

[2] Bengtsson J., Bullock J.M., Egoh B., Everson C., Everson T., O’Connor T., O’Farrell P.J., Smith H.G., Lindborg R. (2019). Grasslands—More Important for Ecosystem Services than You Might Think. Ecosphere.

[3] Dal Prà A., Valli L., Davolio R., Pacchioli M.T., De Monte A., Scotti C. Permanent Meadows and Climate Change in Dairy System Areas in Emilia-Romagna (Italy). Proceedings of the Meeting the Future Demands for Grassland Production.

[4] Klaus V.H., Gilgen A.K., Lüscher A., Buchmann N. (2019). Can We Deduce General Management Recommendations from Biodiversity-Ecosystem Functioning Research in Grasslands?. Res. Collect..

[5] De Deyn G.B., Quirk H., Oakley S., Ostle N.J., Bardgett R.D. (2012). Increased Plant Carbon Translocation Linked to Overyielding in Grassland Species Mixtures. PLoS ONE.

[6] European Commission (1992). Council Directive 92/43 CEE on the conservation of natural habitats and of wild fauna and flora. OJL.

[7] European Commission DG Environment (2007). Interpretation Manual of European Union Habitats (Version EUR27).

[8] Lomba A., Guerra C., Alonso J., Honrado J.P., Jongman R., McCracken D. (2014). Mapping and Monitoring High Nature Value Farmlands: Challenges in European Landscapes. J. Environ. Manag..

[9] Plieninger T., Torralba M., Hartel T., Fagerholm N. (2019). Perceived Ecosystem Services Synergies, Trade-Offs, and Bundles in European High Nature Value Farming Landscapes. Landsc. Ecol..

[10] Perrino E.V., Musarella C.M., Magazzini P. (2021). Management of grazing Italian river buffalo to preserve habitats defined by Directive 92/43/EEC in a protected wetland area on the Mediterranean coast: Palude Frattarolo, Apulia, Italy. Euro-Mediterr. J. Environ. Integr..

[11] Wagensommer R.P., Bartolucci F., Fiorentino M., Licht W., Peccenini S., Perrino E.V., Venanzoni R. (2017). First record for the flora of Italy and lectotypification of the name Linum elegans (Linaceae). Phytotaxa.

[12] Gaucherand S., Liancourt P., Lavorel S. (2006). Importance and intensity of competition along a fertility gradient and across species. J. Veg. Sci..

[13] Gibson C.W.D., Brown V.K. (1991). The effects of grazing on local colonisation and extinction during early succession. J. Veg. Sci..

[14] PNDR-2014-2020-Versiunea-IX-Aprobata-23-Ianuarie-2019.Pdf. https://www.madr.ro/docs/dezvoltare-rurala/2019/PNDR-2014-2020-versiunea-IX-aprobata-23-ianuarie-2019.pdf.

[15] Krautzer B., Gaier L., Weber J., Graiss W., Klingler A. Establishment of Herbs in Species-Poor Grassland. Proceedings of the Meeting the Future Demands for Grassland Production.

[16] Török P., Janišová M., Kuzemko A., Rūsiņa S., Dajić Stevanović Z., Squires V.R., Dengler J., Hua L., Feng H. (2018). Grasslands, Their Threats and Management in Eastern Europe. Grasslands of the World: Diversity, Management and Conservation.

[17] Sutcliffe L., Larkham K., Knowles B. Monitoring High Nature Value Grassland in Transylvania, Romania. Mountain Hay Meadows: Hotspots of Biodiversity and Traditional Culture.

[18] Rūsiņa S., Lakovskis P., Elferts D., Gustiņa L., Dūmiņa I., Kupča L. The Role of Agri-Environmental Policy in the Current Trajectories of Semi-Natural Grassland Management in Latvia. Proceedings of the Meeting the Future Demands for Grassland Production.

[19] Petrescu-Mag R.M., Petrescu D.C., Azadi H., Petrescu-Mag I.V. (2018). Agricultural Land Use Conflict Management—Vulnerabilities, Law Restrictions and Negotiation Frames. A Wake-up Call. Land Use Policy.

[20] Peeters A., Beaufoy G., Canals R.M., De Vliegher A., Huyghe C., Isselstein J., Jones J., Kessler W., Kirilovsky D., Van Den Pol-Van Dasselaar A. Grassland Term Definitions and Classifications Adapted to the Diversity of European Grassland-Based Systems. Proceedings of the 25th EGF General Meeting on “EGF at 50: The Future of European Grasslands.

[21] Krautzer B., Pötsch E.M. The Use of Semi-Natural Grassland as Donor Sites for the Restoration of High Nature Value Areas. Proceedings of the 15th European Grassland Federation Symposium.

[22] ÓhUallacháin D., Sheridan H., Keosh B., Finn J.A. (2018). Agri-Environment Measures for Grassland Habitats: Halting the Decline of Biodiversity, or a Missed Opportunity. Sustainable Meat and Milk Production from Grasslands, Proceedings of the 27th General Meeting of the European Grassland Federation, Cork, Ireland, 17–21 June 2018.

[23] García-de-la-Fuente L., Guzmán Otano D., Mora Cabello de Alba A., Nobre S., Brau-Nogué C. Designing Economic Instruments to Maintain and Enhance Hay Meadows Biodiversity in South-West Europe. Proceedings of the 27th General Meeting of the European Grassland Federation.

[24] Miralles M.T., Särkelä A.K., Koppelmäki K., Tuomisto H., Herzon I. Contribution of High Nature Value farming areas to sustainable livestock production: A pilot case in Finland. Proceedings of the 28th General Meeting of the European Grassland Federation.

[25] Höft A., Müller J., Gerowitt B. (2010). Vegetation Indicators for Grazing Activities on Grassland to Be Implemented in Outcome-Oriented Agri-Environmental Payment Schemes in North-East Germany. Ecol. Indic..

[26] Melts I., Lanno K., Sammul M., Uchida K., Heinsoo K., Kull T., Laanisto L. (2018). Fertilising Semi-Natural Grasslands May Cause Long-Term Negative Effects on Both Biodiversity and Ecosystem Stability. J. Appl. Ecol..

[27] Curth-van Middelkoop J.C., de Boer H.C., Galama P.J. (2020). Characteristics of Organic Manure from’Freewalk’housing, Compared with Slurry, and Their Appreciation by Farmers. Meeting the Future Demands for Grassland Production: Proceedings of the 28th General Meeting of the European Grassland Federation, Helsinki, Finland, 19–22 October 2020.

[28] Samuil C., Vintu V., Popovici C.I., Stavarache M. (2014). Influence of Fertilization on the Biodiversity of *Festuca rubra* L. and *Agrostis capillaris* L. Grassland. Future Eur. Grassl..

[29] Coldea G., Oprea A., Sârbu I., Sârbu C., Ștefan N. (2012). Les Associations Vegetal de Romaine.

[30] Cojocariu L., Copăcean L., Popescu C. (2019). Conservation of grassland habitats biodiversity in the context of sustainable development of mountain area of Romania. Appl. Ecol. Environ. Res..

[31] Tong Z., Quan G., Wan L., He F., Li X. (2019). The effect of fertilizers on biomass and biodiversity on a semi-arid grassland of Northern China. Sustainability.

[32] Reif A., Ruşdea E., Păcurar F., Rotar I., Brinkmann K., Auch E., Goia A., Bühler J. (2008). A traditional cultural landscape in transformation. Mt. Res. Dev..

[33] Bohner A. (2005). Soil Chemical Properties as Indicators of Plant Species Richness in Grassland Communities. Grassl. Sci. Eur..

[34] Carnus J.-M., Parrotta J., Brockerhoff E., Arbez M., Jactel H., Kremer A., Lamb D., O’Hara K., Walters B. (2006). Planted Forests and Biodiversity. J. For..

[35] Ionescu I., Osiceanu M. (2007). The Floristic Biodiversity of the Main Hill and Mountain Pasture Types from the SW of Romania and Their Productive Capacity. Permanent and Temporary Grassland: Plant, Environment and Economy, Proceedings of the 14th Symposium of the European Grassland Federation, Ghent, Belgium, 3–5 September 2007.

[36] Mosquera-Losada M.R., Rigueiro-Rodríguez A. (2014). Agroforestry Systems: An Option for Mitigation and Adaptation to Overcome Global Climate Change. Future Eur. Grassl..

[37] Grabowski K., Grzegorczyk S., Olszewska M., Lachacz A. (2020). The Effect of Long-Term Wastewater Irrigation on the Botanical Composition of Meadow Sward, Yield and Nutritional Value of Hay. J. Elem..

[38] Garda N. (2010). The Study of Some Mountainous Landscape Elements (With Special Regard to Grassland Ecosystems in Gârda de sus Commune, Apuseni Mountains). Ph.D. Thesis.

[39] Vintu V., Samuil C., Popovici I.C., Saghin G. (2010). Improving Grasslands of *Agrostis capillaris* and *Festuca rubra* in the Carpathian Mountains of Romania by Organic Fertilization. Grassl. A Chang. World.

[40] Stukonis V. (2005). Central Lithuania’s Natural Grasslands and Their State. Integr. Effic. Grassl. Farming Biodivers..

[41] Stoie A., Păcurar F., Rotar I., Parte I. (2007). Studies regarding the Arnica montana meadows from the central part of the Apuseni Mountains. Scientifical Papers of Agriculture XXXIX.

[42] Musial K., Szewczyk W., Walczak J., Grygierzec B. (2017). The Role of Re-Introducing Sheep Grazing on Protected Calcareous Xerothermic Grassland. Grassl. Sci. Eur..

[43] Ronch F., da Macolino S., Ziliotto U. (2007). Effects of Different Periods of Abandonment on the Features of the Class Festuco-Brometea Br.-Bl. Meadows in NE Italy. Permanent and Temporary Grassland: Plant, Environment and Economy. Proceedings of the 14th Symposium of the European Grassland Federation, Ghent, Belgium, 3–5 September 2007.

[44] Huguenin-Elie O., Stutz C.J., Gago R., Lüscher A. (2010). Restoration of Species-Rich Grasslands: Reduction in Nutrient Availability Slightly Improved Forb Species’ Establishment. Grassl. A Chang. World.

[45] Vaida I. (2018). The Influence of Management on the Agronomic and Ecological Value of Mountain Grasslands. Ph.D. Thesis.

[46] Rotar I., Vaida I., Păcurar F. (2020). Species with Indicative Values for the Management of the Mountain Grasslands. Rom. Agric. Res. Nardi Fundulea.

[47] Vintu V., Samuil C., Sirbu C., Popovici C.I., Stavarache M. (2011). Sustainable Management of *Nardus stricta* L. Grasslands in Romaniaâ€^TM^ s Carpathians. Not. Bot. Horti Agrobot. Cluj-Napoca.

[48] Samuil C., Stavarache M., Sirbu C., Vintu V. (2018). Influence of Sustainable Fertilization on Yield and Quality Food of Mountain Grassland. Not. Bot. Horti. Agrobot. Cluj-Napoca.

[49] Gląb T., Kacorzyk P. (2011). Root Distribution and Herbage Production under Different Management Regimes of Mountain Grassland. Soil Tillage Res..

[50] Pykälä J. (2000). Mitigating Human Effects on European Biodiversity through Traditional Animal Husbandry. Conserv. Biol..

[51] Coldea G., Filipaş L., Fărcaş S., Stoica A., Ursu T., Pop A.-M. (2008). The Relationship between the Structure of Grasslands from the North-Eastern Slope of the Vlădeasa Massif and Socio-Economic Activities. Contrib. Bot..

[52] Andrey A., Humbert J.-Y., Pernollet C., Arlettaz R. (2014). Experimental Evidence for the Immediate Impact of Fertilization and Irrigation upon the Plant and Invertebrate Communities of Mountain Grasslands. Ecol. Evol..

[53] Viebranz K. (2015). Comparison of Plant Species Communities in Meadows of the Nature Reserve “Bodenmöser” from 1987 to 2014. Master’s Thesis.

[54] Walcher R., Hussain R.I., Sachslehner L., Bohner A., Jernej I., Zaller J.G., Arnberger A., Frank T. (2019). Long-Term Abandonment of Mountain Meadows Affects Bumblebees, True Bugs and Grasshoppers: A Case Study in the Austrian Alps. Appl. Ecol. Environ. Res..

[55] Cirebea M., Rotar I., Vidican R., Pleșa A., Morea A., Ranta O. (2020). Impact of Organo-Mineral Fertilization upon Phytocoenosis and Feed Quality of the Grasslands in the Region of Transylvania. Rom. Agric. Res..

[56] Škodová I., Devánová K., Senko D. (2011). Subxerophilous and Mesophilous Grasslands of the Biele Karpaty Mts. (White Carpathian Mts.) in Slovakia. Tuexenia.

[57] Krause B., Culmsee H. (2013). The Significance of Habitat Continuity and Current Management on the Compositional and Functional Diversity of Grasslands in the Uplands of Lower Saxony, Germany. Flora-Morphol. Distrib. Funct. Ecol. Plants.

[58] Dmytrash-Vatseba I.I., Shumska N.V. (2020). Dynamics of Plant Cover of Meadow Steppes after the Cessation of Traditional Management in Opillia. Biosyst. Divers..

[59] Păcurar F., Balazsi A., Rotar I., Vaida I., Reif A., Vidican R., Rușdea E., Stoian V., Sangeorzan D. (2020). Technologies Used for Maintaining Oligotrophic Grasslands and Their Biodiversity in a Mountain Landscape. Rom. Biotechnol. Lett..

[60] Cipriotti P.A., Rauber R.B., Collantes M.B., Braun K., Escartín C. (2010). Hieracium Pilosella Invasion in the Tierra Del Fuego Steppe, Southern Patagonia. Biol. Invasions.

[61] Díaz-Barradas M.C., Zunzunegui M., Álvarez-Cansino L., Esquivias M.P., Collantes M.B., Cipriotti P.A. (2015). Species-Specific Effects of the Invasive Hieracium Pilosella in Magellanic Steppe Grasslands Are Driven by Nitrogen Cycle Changes. Plant Soil.

[62] Zarzycki J., Kopeć M. (2020). The Scheme of Nutrient Addition Affects Vegetation Composition and Plant Species Richness in Different Ways: Results from a Long-Term Grasslands Experiment. Agric. Ecosyst. Environ..

[63] French K., Watts E. (2020). Differences in Vegetative Growth of Two Invasive Hawkweeds at Temperatures Simulating Invaded Habitats at Two Altitudes. Sci. Rep..

[64] Van Dobben H.F., Wamelink G.W., Slim P.A., Kamiński J., Piórkowski H. (2017). Species-Rich Grassland Can Persist under Nitrogen-Rich but Phosphorus-Limited Conditions. Plant Soil.

[65] Hejcman M., Schellberg J., Pavluu V. (2010). Long-Term Effects of Cutting Frequency and Liming on Soil Chemical Properties, Biomass Production and Plant Species Composition of Lolio-Cynosuretum Grassland after the Cessation of Fertilizer Application. Appl. Veg. Sci..

[66] Hejcman M., Sochorová L., Pavluu V., Štrobach J., Diepolder M., Schellberg J. (2014). The Steinach Grassland Experiment: Soil Chemical Properties, Sward Height and Plant Species Composition in Three Cut Alluvial Meadow after Decades-Long Fertilizer Application. Agric. Ecosyst. Environ..

[67] Mládková P., Mládek J., Hejduk S., Hejcman M., Cruz P., Jouany C., Pakeman R.J. (2015). High-Nature-Value Grasslands Have the Capacity to Cope with Nutrient Impoverishment Induced by Mowing and Livestock Grazing. J. Appl. Ecol..

[68] Roleček J., Dřevojan P., Hájková P., Hájek M. (2019). Report of New Maxima of Fine-Scale Vascular Plant Species Richness Recorded in East-Central European Semi-Dry Grasslands. Tuexenia.

[69] Păcurar F., Rotar I., Vaida I., Vidican R., Mălinaş A. (2017). Indicator Species of Fertilization Intensity in Mountain Grasslands. Grassland resources for extensive farming systems in marginal lands: Major drivers and future scenarios. Grassl. Sci. Eur..

[70] Pavlu V., Gaisler J., Pavlu L., Hejcman M., Ludvíková V. (2012). Effect of Fertiliser Application and Abandonment on Plant Species Composition of *Festuca rubra* Grassland. Acta Oecol..

[71] Lawniczak A., Drapikowska M., Celka Z., Szkudlarz P., Jackowiak B. (2011). Response of Anthoxanthum Odoratum and A. Aristatum to the Different Habitat Types and Nutrient Concentration in Soil. Fresen. Environ. Bull..

[72] Enri S.R., Nucera E., Lonati M., Alberto P.F., Probo M. (2020). The Biodiversity Promotion Areas: Effectiveness of Agricultural Direct Payments on Plant Diversity Conservation in the Semi-Natural Grasslands of the Southern Swiss Alps. Biodivers. Conserv..

[73] Winter S., Penker M., Kriechbaum M. (2011). Integrating Farmers’ Knowledge on Toxic Plants and Grassland Management: A Case Study on Colchicum Autumnale in Austria. Biodivers. Conserv..

[74] Swallow K.A., Wood M.J., Goodenough A.E. (2020). Relative Contribution of Ancient Woodland Indicator and Non-Indicator Species to Herb Layer Distinctiveness in Ancient Semi-Natural, Ancient Replanted, and Recent Woodland. Appl. Veg. Sci..

[75] Mardari C. (2012). Vegetal Communities of Juncetea Trifi Di Hadač 1946 from the Hydrographic Basin of Neagra Broştenilor River (Romania, Eastern Carpathians). Conserv. Plant Divers..

[76] Mardari C., Dănilă D., Bîrsan C., Balaeş T., Ştefanache C., Tănase C. (2015). Plant Communities with Arnica Montana in Natural Habitats from the Central Region of Romanian Eastern Carpathians. J. Plant Dev..

[77] Rowland D. (2012). Mechanisms of Invasion of Hieracium Aurantiacum and Leucanthemum Vulgare in Kosciuszko National Park. Ph.D. Thesis.

[78] Pruchniewicz D. (2017). Abandonment of Traditionally Managed Mesic Mountain Meadows Affects Plant Species Composition and Diversity. Basic Appl. Ecol..

[79] Birkhofer K., Rusch A., Andersson G.K., Bommarco R., Dänhardt J., Ekbom B., Jönsson A., Lindborg R., Olsson O., Rader R. (2018). A Framework to Identify Indicator Species for Ecosystem Services in Agricultural Landscapes. Ecol. Indic..

[80] Schmid B.C., Poschlod P., Prentice H.C. (2017). The Contribution of Successional Grasslands to the Conservation of Semi-Natural Grasslands Species–A Landscape Perspective. Biol. Conserv..

[81] Wagner M., Fagan K.C., Jefferson R.G., Marrs R.H., Mortimer S.R., Bullock J.M., Pywell R.F. (2019). Species Indicators for Naturally-Regenerating and Old Calcareous Grassland in Southern England. Ecol. Indic..

[82] Babai D., Tóth A., Szentirmai I., Biró M., Máté A., Demeter L., Szépligeti M., Varga A., Molnár Á., Kun R. (2015). Do Conservation and Agri-Environmental Regulations Effectively Support Traditional Small-Scale Farming in East-Central European Cultural Landscapes?. Biodivers. Conserv..

[83] Streitberger M., Schmidt C., Fartmann T. (2017). Contrasting Response of Vascular Plant and Bryophyte Species Assemblages to a Soil-Disturbing Ecosystem Engineer in Calcareous Grasslands. Ecol. Eng..

[84] Adamczyk J., Krysiak S., Adamczyk J. (2016). Diversity of the flora and vegetation of abandoned farmlands. The Ecological Role of Abandoned Agricultural Lands in Buffer Zones Around Landscape Parks in the Lódź Voivodeship.

[85] Hejcman M., Schellberg J., Pavlu V. (2011). Competitive Ability of *Rhinanthus minor* L. in Relation to Productivityin the Rengen Grassland Experiment. Plant Soil Environ..

[86] Siehoff S., Lennartz G., Heilburg I.C., Roß-Nickoll M., Ratte H.T., Preuss T.G. (2011). Process-Based Modeling of Grassland Dynamics Built on Ecological Indicator Values for Land Use. Ecol. Model..

[87] Ellenberg H. (1991). Zeigerwerte von Pflanzen in Mitteleuropa. Scr. Geobot..

[88] Ellenberg H., Weber H.E., Düll R., Wirth V., Werner W., Paulißen D. (1992). Zeigerwerte von Pflanzen in Mitteleuropa. Scripta Geobotanica. GöttingenGoltze.

[89] Dierschke H., Briemle G. (2002). Kulturgrasland: Wiesen, Weiden Und Verwandte Staudenfluren.

[90] Oroian S., Sămărghițan M., Hirițiu M., Coșarcă S., Tanase C. (2016). High Nature Value Grasslands from Arrhenatherion Alliance Identified in Mureș County. Bull. Univ. Agric. Sci. Vet. Med. Cluj-Napoca Agric..

[91] Duffková R., Libichová H. (2013). Effects of Cattle Slurry Application on Plant Species Composition of Moderately Moist Arrhenatherion Grassland. Plant Soil Environ..

[92] Paulisová M., Vozár L., Kovár P., Hric P., Verešová P. (2019). Changes in Floristic Composition of Grassland Affected by the Different Exploitation Intensity. Acta Fytotechn. Zootech..

[93] McCouch S., Baute G., Bradeen J., Bramel P., Bretting P., Buckler E., Burke J., Charest D., Cloutier S., Cole G. (2013). Agriculture: Feeding the future. Nature.

[94] Maxted N., Ford-Lloyd B.V., Jury S.L., Kell S.P., Scholten M.A. (2006). Towards a definition of a crop wild relative. Biodivers. Conserv..

[95] Gillet F., Mauchamp L., Badot P.-M., Mouly A. (2016). Recent Changes in Mountain Grasslands: A Vegetation Resampling Study. Ecol. Evol..

[96] Busch V., Klaus V.H., Schäfer D., Prati D., Boch S., Müller J., Chisté M., Mody K., Blüthgen N., Fischer M. (2019). Will I Stay or Will I Go? Plant Species-Specific Response and Tolerance to High Land-Use Intensity in Temperate Grassland Ecosystems. J. Veg. Sci..

[97] Maruşca T., Mocanu V., Haş E.C., Tod M.A., Andreoiu A.C., Dragoş M.M., Blaj V.A., Ene T.A., Silistru D., Ichim E. (2014). Ghid de Întocmire a Amenajamentelor Pastorale. Braşov. Capolav. Rom..

[98] Dillon P.G. (2018). The Evolution of Grassland in the European Union in Terms of Utilisation, Productivity, Food Security and the Importance of Adoption of Technical Innovations in Increasing Sustainability of Pasture-Based Ruminant Production Systems. Sustain. Meat Milk Prod. Grassl..

[99] Lind V., Mølmann J. (2011). Effects of Feed Quality of Mountain Pastures and Cultivated Pastures on Lamb Meat Quality in Norway. Proceedings of the Grassland farming and land management systems in mountainous regions. In Proceedings of the 16th Symposium of the European Grassland Federation, Gumpenstein, Austria, 29–31 August 2011.

[100] Pavlu L., Poetsch E.M., Pavlu V., Hejcman M., Hujerová R., Gaisler J. (2016). Effect of Different N, P, K Fertilisation on Plant Species Composition and Species Richness in an Alluvial Meadow. Multiple Roles of Grassland in the European Bioeconomy, Proceedings of the 26th General Meeting of the European Grassland Federation, Trondheim, Norway, 4–8 September 2016.

[101] Păcurar F., Rotar I., Albert R., Vidican R., Stoian V., Gaertner S.M., Allen R.B. (2014). Impact of Climate on Vegetation Change in a Mountain Grassland-Succession and Fluctuation. Not. Bot. Horti Agrobot. Cluj-Napoca.

[102] Păcurar F., Rotar I. (2014). Metode de Studiu Şi Interpretare a Vegetaţiei Pajiştilor.

[103] De Mendiburu F., de Mendiburu M.F. (2019). Package ‘Agricolae’. R PackageVersion.

[104] R Core Team (2020). A Language and Environment for Statistical Computing.

[105] McCune B., Grace J.B., Urban D.L. (2002). Analysis of Ecological Communities.

[106] Legendre P., Legendre L. (2012). Numerical Ecology.

[107] Kent M. (2011). Vegetation Description and Data Analysis: A Practical Approach.

[108] Peck J.E. (2010). Multivariate Analysis for Community Ecologists.

