# Supplementary material for: Changes in Diversity Due to Long-Term Management in a High Natural Value Grassland
Source: Plants (Basel). 2021 Apr 9;10(4):739. doi: 10.3390/plants10040739 (PMC8069202; doi:10.3390/plants10040739)
Supplement: Supplementary file 1 [file plants-10-00739-s001.pdf]

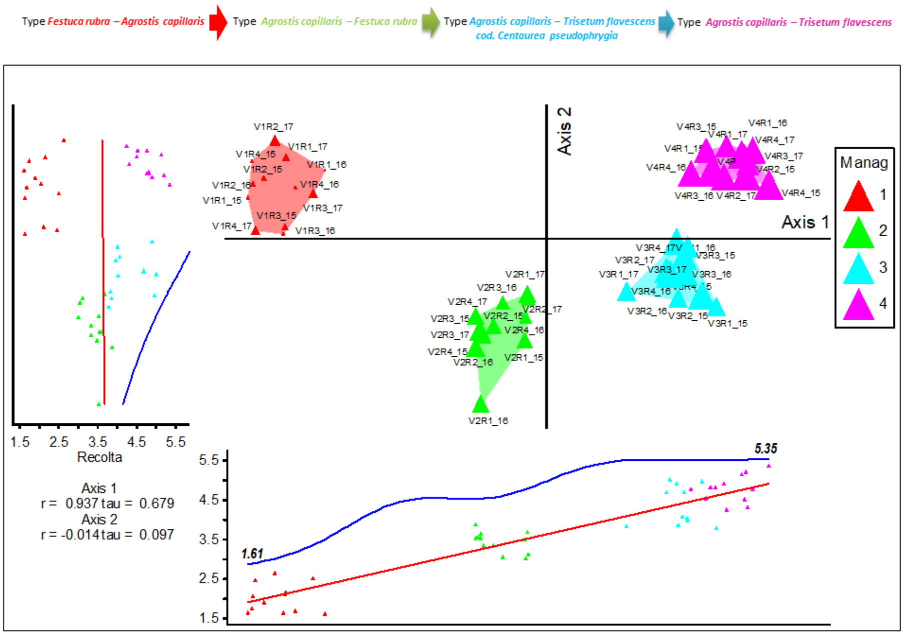

Figure S1. The influence of organic fertilizer on the dry matter (DM) yield

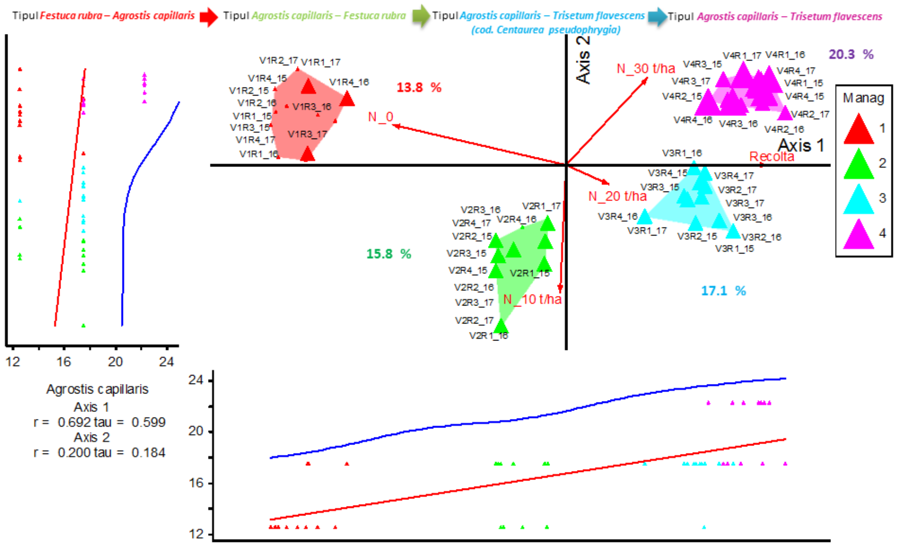

Figure S2. The influence of *Agrostis capillaris* species on the dry matter (DM) yield

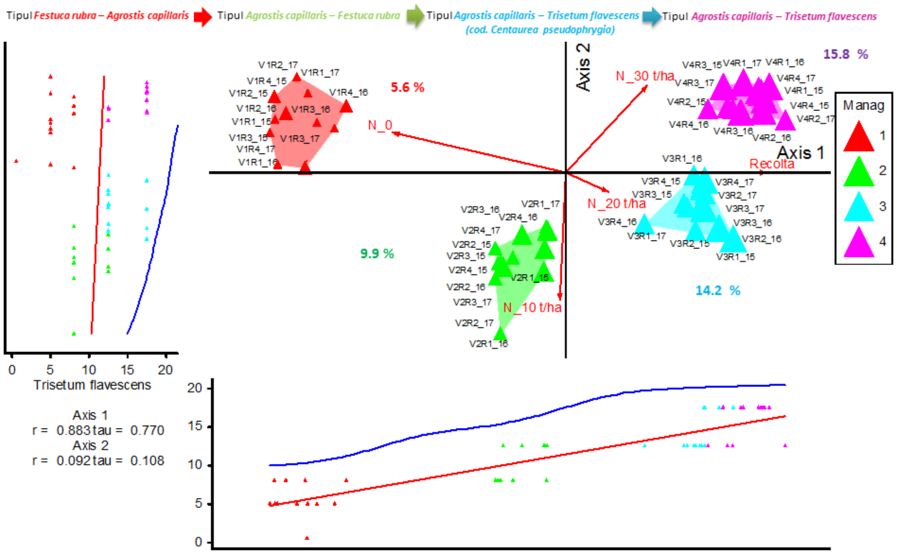

Figure S3. The influence of *Trisetum flavescens* species on dry matter (DM) yield

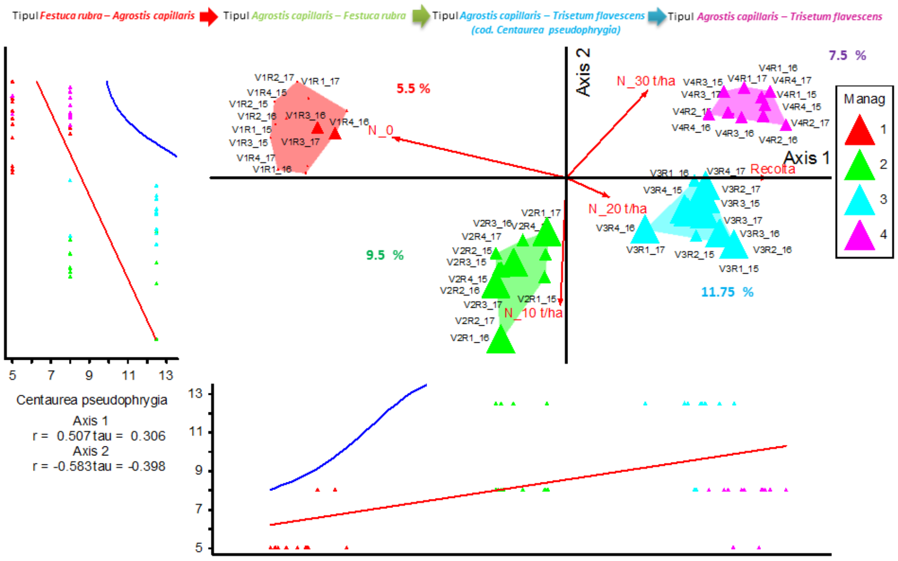

Figure S4. The influence of *Centaurea pseudophrygia* species on the dry matter (DM) yield

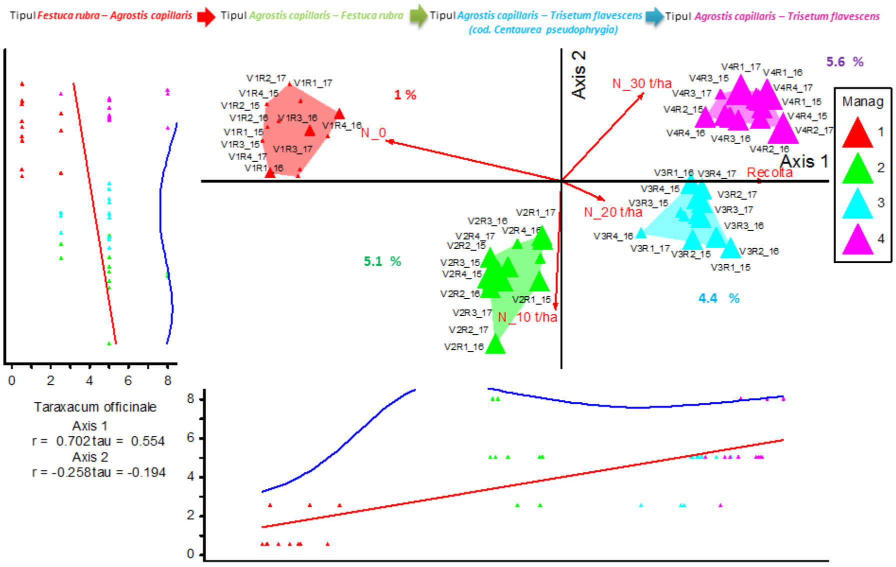

Figure S5. The influence of *Taraxacum officinale* species on the dry matter (DM) yield

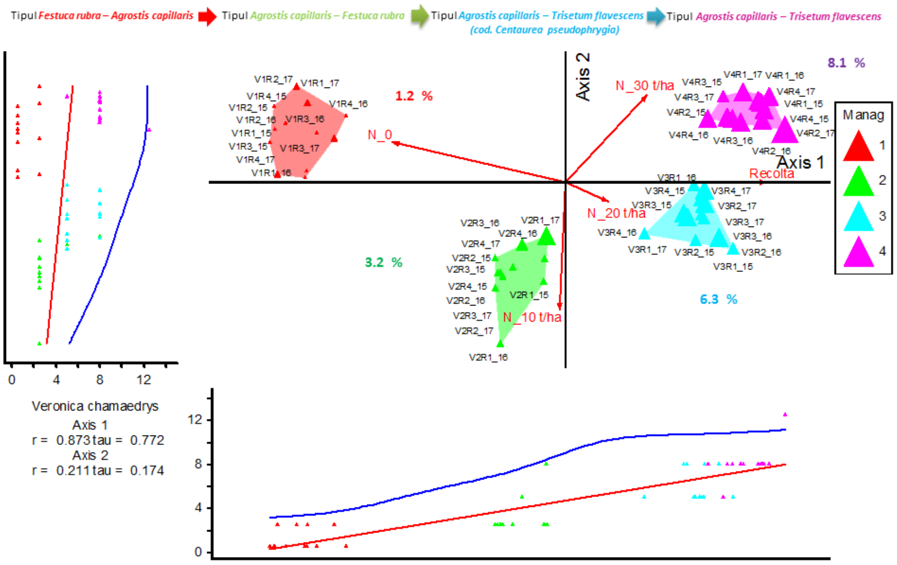

Figure S6. The influence of *Veronica chamaedrys* species on the dry matter (DM) yield
